# Impact of an online learning by concordance program on reflection

**DOI:** 10.1186/s12909-023-04799-9

**Published:** 2023-11-01

**Authors:** Léa Charton, Abdelkader Lahmar, Elodie Hernandez, Fabien Rougerie, Mathieu Lorenzo

**Affiliations:** 1 Département de Médecine Générale et de la Formation Territoriale, Faculté de Médecine, Maïeutique et Sciences de la Santé, 4 rue Kirschleger, Strasbourg, 67085 France; 2 Département de Médecine Générale, Faculté de Médecine, Besançon, France

**Keywords:** Reflection, Learning by concordance, Clinical reasoning, Assessment, ECG interpretation

## Abstract

**Background:**

Learning by concordance (LbC) is a recent approach that introduces learners to the complexity and uncertainty of clinical practice. Some data on LbC suggest that it stimulates reflection in future clinicians. We developed an online LbC training program on electrocardiogram (ECG) interpretation in general practice at the University of Strasbourg, France, and conducted an exploratory qualitative study to document the impact of this ECG learning-by-concordance training program on reflection in participants.

**Methods:**

We created 18 clinical vignettes on ECG interpretation based on a review of the literature on frequent and serious cardiovascular diseases that can be identified using an ECG in general practice. The training program was delivered online to postgraduate general practice students in two faculties of medicine. We conducted a qualitative study based on thematic analysis of two focus groups and six individual interviews. Inductive and deductive coding were performed. The five major components of reflection in the Nguyen model were used in the deductive coding: (i) thoughts and actions, (ii) attentive, critical, exploratory, and iterative processes (ACEI), (iii) underlying conceptual frame, (iv) change and (v) self.

**Results:**

Two focus groups and six individual interviews were conducted. The qualitative analysis indicated 203 codes in the focus groups and 206 codes in the individual interviews, which were divided into five groups based on the components of reflection in the Nguyen model: (i) the self; (ii) attentive, critical, exploratory, and iterative interactions with (iii) one’s thoughts and actions; and (iv) a view on both the change itself and (v) the underlying conceptual frame. Inductive coding revealed interesting insights into the impact of the identity of the panel members, the absence of a scoring system and the question of uncertainty in ECG reading.

**Conclusions:**

This study supports the claim that the use of LbC in the context of ECG interpretation could foster reflection in future general practitioners. We discuss future research avenues on instructional design of LbC and reflection.

**Supplementary Information:**

The online version contains supplementary material available at 10.1186/s12909-023-04799-9.

## Background

Learning by concordance (LbC) is a fairly recent approach that introduces learners to the complexity and uncertainty of clinical practice [1–4]. The most well-known format of LbC is the script concordance test, which has broadly been recognized as a useful tool for assessing clinical reasoning [2, 5]. In the past 5 years, other LbC formats have been described with more emphasis on the justification of reasoning than on the results of such reasoning [1, 2]. As Charlin et al. stated in 2021 [2], LbC is “*an on-line educational strategy that makes learners practice reasoning competency in case-based clinical situations. The questions asked are similar to those professionals ask themselves in their practice and participant answers are compared to those of a reference panel. When participants answer the questions, they receive an automated feedback that is two-fold as they see how the panelists respond and justifications each panelist gives for their answer. This provides rich contextual knowledge about the situation, supplemented by a synthesis summarizing crucial points”* [2].

Fernandez et al. suggested in 2016 that LbC stimulate reflection in future clinicians [1]. The authors relied on the analysis of open-ended comments from a sample of students who participated in an LbC program [1]. This claim appears methodologically fragile and requires further substantiation [6]. The same team recently published a new study on the design of LbC [7]. Based on dialogue-group sessions with eight clinical educators, the authors argue about the impact of pedagogical choices regarding LbC design on students’ reflection [7]. To the best of our knowledge, there was no other data in the literature regarding the impact of LbC on students’ reflection.

Reflection is regarded by many as an essential characteristic for professional competence [8]. It could contribute to improving their performance [9–12]. According to Nguyen et al., reflection is therefore defined as “the process of engaging the self in attentive, critical, exploratory and iterative interactions with one’s thoughts and actions, and their underlying conceptual frame, with a view to changing them and a view on the change itself” [9]. Other definitions of reflection have been used in medical education for nearly one century. Definitions of reflection provided by preeminent authors such as Dewey [13] or Schön [14] have been widely employed. However, as reflection is a complex construct, some authors have argued that using a common, explicit understanding of reflection could help teaching and research projects on this topic. The definition and model of reflection provided by Nguyen et al. are now commonly used in the medical education community [15].

Electrocardiogram (ECG) interpretation is a simple paraclinical test that aims to detect heart diseases. The ECG interpretation performance of both medical students and doctors is poor, which may impact patient outcomes [16, 17]. Interventions such as checklists intended to help learners have not been reported to have an overall effect in reducing diagnostic errors in ECG interpretation [18]. No single approach to or format for ECG teaching seems to be superior to other approaches or formats [19]. However, self-directed learning seems to be associated with poorer interpretation performance, and computer-based learning may be advantageous [19].

We developed an online LbC training program on electrocardiogram (ECG) interpretation in general practice at the University of Strasbourg, France. We conducted an exploratory study to document the impact of this ECG learning-by-concordance training program on reflection in participants.

## Methods

### Context

This study took place within the medical faculties of Strasbourg and Besançon in France. These faculties enroll approximately 200 students in their third-cycle general medicine programs. The authors of this study serve as educators within these faculties.

### ECG LbC design

LC and ML conducted a review of the literature on the frequent and serious cardiovascular diseases that can be identified using an ECG in general practice. Twenty-four clinical vignettes pertaining to these diseases as well as two vignettes featuring a normal ECG were drafted by LC, FR and another experienced family physician. These vignettes were first submitted to a seven-member panel at the general practice department (GPD) of the University of Strasbourg’s Faculty of Medicine. The panel was asked to assess the authenticity of the vignettes on a Likert scale ranging from 0 (no authenticity) to 4 (maximal authenticity) and to suggest possible changes. Two vignettes with an authenticity score below 2.5/4 were removed, and two other vignettes were rewritten according to suggestions made by members of the panel.

The remaining vignettes were submitted to a panel of eight volunteer cardiologists who completed their interpretation of the ECG based on the clinical vignette they were provided. Two vignettes were removed at that stage due to significant disagreements in the interpretation of the ECG strips.

Four members of the Strasbourg GPD who had access to the cardiologists’ ECG interpretations then drafted care proposals. Two vignettes were removed at that stage due to significant disagreements in the proposals.

Finally, FR, who had expertise in the use of ECGs in general practice, drafted a synthesis and listed bibliographic references pertaining to the theme of the vignette.

The training program in its final form comprised eighteen clinical vignettes. For each, a textual clinical vignette was presented to learners, which focused on a real-life situation involving an ECG. The learners were asked to interpret the ECG and to formulate diagnostic hypotheses concerning the clinical situation. Once this step was successfully completed, they accessed a second page where they were asked to compare their interpretation and hypotheses with those offered by a panel of expert cardiologists and to produce a patient care proposal while taking the opinions of the expert cardiologists into consideration. On the third page, they compared their care proposal with those made by a panel of expert family doctors. Finally, they were asked to read a synthesis of the case that was drafted by a family physician with expertise in the field of ECG interpretation, which included links to bibliographical references. One example of a vignette can be found in supplementary material.

One of the eighteen vignettes was used as a tutorial at the beginning of the training, including suggestions for responses and advice regarding how to use the expert panel feedback. The goal of this tutorial was to address difficulties in the appropriation of LbC as described in the literature [2, 4].

Unlike the LbC tools that have previously been described in the literature [2–4], no score was calculated. This program was purely training-oriented and aimed at improving the knowledge of students on situations involving an ECG as well as encouraging reflection. We therefore chose to not provide any score. We did not display the identities of panel members to the learners, as we did not receive their permission to do so.

Approximately one hundred students have been undergoing this LbC training program each year since 2018.

### Selection of expert panels

The panel of cardiologists was assembled by randomly contacting thirty independent and hospital-affiliated cardiologists in the Alsace and Rhône-Alpes regions of France. Eight cardiologists agreed to participate in the study.

The panel of family physicians was assembled on a voluntary basis from the twelve members of the GPD with expertise in ECG interpretation. Four physicians agreed to participate in the study.

### Participants

The training program was offered online to all postgraduate general practice students at the University of Strasbourg’s Faculty of Medicine and the University of Besançon’s Faculty of Medicine (both in France). Students were free to complete the training when they wished over a period of three months. In the winter of 2018, students who had completed the training received an email invitation to participate in focus groups as part of a study exploring the learning outcomes of the training. 38 students who volunteered to participate were sent a survey to determine a mutually convenient date for scheduling the focus group sessions. Students available during the same time slot were included in the focus groups. The focus groups were then supplemented by individual interviews.

### Qualitative study of reflection

A qualitative study was conducted based on thematic analysis of two focus groups and six individual interviews convened in the Faculties of Medicine of the Universities of Strasbourg and Besançon. We chose focus groups to encourage participants to confront their points of view regarding the impact of the LbC training program on reflection and the identification of convergent opinions [20]. We then conducted individual interviews to obtain a better understanding of the underlying reflective processes, which are more difficult to access spontaneously from the individual’s consciousness [15].

EH and AL were junior lecturers in general practice at the Medical Schools of Besançon (EH) and Strasbourg (AL). EH conducted the focus groups, and AL conducted the individual interviews. Both researchers were trained in qualitative research and had previously been involved in focus groups and individual interviews both as participants and investigators. They maintained a logbook throughout the research process.

EH knew some of the participants in the Medical School of Besançon. She specified her role within the framework of the focus group and highlighted the distinction between her roles as a researcher and a teacher. The age difference between the participants and the investigator was small. A scribe whom the participants did not know participated only in the second focus group. AL did not know any of the participants.

Participants in the focus groups and individual interviews were only aware of the general theme of the research. The only criteria for inclusion were having completed the training program and volunteering to participate. There were no exclusion criteria. The investigators presented the research by explaining the qualitative method and the preservation of the anonymity of participants; they also secured participants’ oral consent for the audio recording and transcription of the focus group and individual interviews.

Students who were available to participate during the proposed dates were contacted via email. Data were collected from the focus group in a restaurant in April 2018 (first focus group) and in the University of Besançon’s Faculty of Medicine in May 2018 (second focus group). Data were collected from the individual interviews in the Faculty of Medicine of the University of Strasbourg between March and September 2022.

The interview guides were prepared beforehand and submitted to a group of qualitative research experts before the data collection phase; however, they were not tested. These guides were refined after the first focus group and the first individual interview. Additional file 2 presents the interview guides.

During the individual interviews, AL projected the LbC program on a wide screen. Each participant could progressively review the training, including their own answers. Vignettes were picked randomly, and the participant was invited to comment on how they progressed through the training program.

The investigators used an audio recording to transcribe the discussions extensively. Field notes were taken before the interviews to describe the participants. The participants were notified that they could ask for the transcripts to make changes or comments, but no participants took advantage of this offer. All the verbatim interviews were anonymized.

EH and AL coded the entirety of the data using a thematic approach with the help of NVivoâ software. Joint coding was performed by ML by reference to a random sample of 15% of transcripts. A 90% consensus on coding was attained through discussion between the authors [21]. Inductive and deductive coding were performed. The five major components of the reflection of the Nguyen model were used during the deductive coding: (i) thoughts and actions, (ii) attentive, critical, exploratory and iterative processes (ACEI), (iii) underlying conceptual frame, (iv) change and (v) self [9]. Data saturation was not sought since we adopted an exploratory perspective as well as for pragmatic considerations, such as participant availability. Inductive coding was inspired by the grounded-theory approach. This approach was used to allow unexpected aspects of the impact of LbC on reflection to emerge from our data.

Data processing concerning the focus groups began in April 2018 with the first focus group and finished in December 2018 when a consensus was reached regarding the coding. Data processing concerning the individual interviews was conducted between September 2022 and January 2023.

## Results

The two focus groups featured a total of ten participants (participant nos. 1 to 10), and six individual interviews were conducted (participant nos. 11 to 16). Table 1 presents the characteristics of the participants. The first focus group lasted 1 h 25 min, and the second lasted 1 h 50 min. The single motive indicated for nonparticipation pertained to scheduling issues. Individual interviews were conducted, which lasted from 30 to 54 min. Table 2 provides an overview of thematic categories and representative quotations for the deductive and inductive coding.

**Table 1:** Characteristics of participants

Participants	Age (years)	Sex	Year of postgraduate training
No. 1	26	Male	2
No. 2	29	Male	3
No. 3	25	Female	1
No. 4	26	Female	1
No. 5	30	Female	3
No. 6	27	Female	3
No. 7	26	Male	2
No. 8	27	Female	2
No. 9	27	Female	3
No. 10	26	Female	1
No. 11	25	Male	1
No. 12	26	Female	1
No. 13	25	Female	1
No. 14	25	Male	1
No. 15	30	Female	3
No.16	25	Male	1

**Table 2:** Thematic categories and representative quotes of deductive and inductive coding

Deductive coding	
Thematic categories	Representative quotes
**“Thoughts and actions” component**	
	*“You realize that there are several possible answers and that it’s no because yours is a bit different that you’ve got it wrong, necessarily*” (participant no. 8).
	“*About the ECG, since it’s normal, I don’t do anything, but obviously you reassure [the patient]. Of course, right? Now [during the internship] obviously you always reassure people, you don’t even realize, actually*” (participant no. 3).
	*“You start to think, really thinking, we don’t just watch the ECG so we know what are the stakes”* (participant no.4)
**ACEI component**	
Attentive process	“*I would put myself in the situation, I’d tell myself, I’m the doctor, this patient is my patient, and yeah, I was really totally in the case*” (participant no. 8)
	*“I really did it like I was the GP”* (participant no.1)
Critical process	*“So, the thing is, it made me ask myself by the end of the 18 cases: have you been dangerous or not?*” (participant no.10)
	*“I was asking myself about the synthesis: is it really what you should do?“* (participant no.5)
Exploratory process	“*You’re going to be judged, they’ll say: “wait a minute, X wrote this, she missed the infarction, she didn’t send him [to the emergency ward] he’d be dead […]”, but the fact that this is completely anonymous [for the participants], I think that for a training program like this it really is very important*” (participant no.3)
	*“(the formation) allowed me to ask myself some questions“(*participant no.1)
Iterative process	*“after doing this again and again (by answering questions in the LbC ECG) […] I understood that in this kind of situation, I could refer to a cardiologist”* (participant no.8)
	“*Well I adjusted to the answers, anyway – for instance, about reassurance there was a first case where I hadn’t written it down, and in the answer, he put reassurance, and so in the last case where it was anxiety and all that, I put reassurance*” (participant n°9)
**Self component**	
	*“it showed me that there is a lot of situations where ECG can help the physician in his practice”* (participant no.2)
	“*I think it isn’t bad, doing it like this, because in the end when I did it I wasn’t under any pressure, and so you know, you’re not stressed out. When you’re being graded or evaluated, you get stressed out, whereas this is really just for us*” (participant no. 5)
**Change component**	
	“*My way of reasoning is fairly logical, compared to the others, I’m a little bit reassured, I’ve gained a little bit of trust in myself*” (participant no. 3)
	“*for instance, you see [atrial fibrillation] […] I told myself, ‘OK, we have the right to handle that ourselves’*” (participant no. 1)
**Underlying conceptual frames component**	
	“*it’s true that for hyperkalemia it’s a typical picture to recognize directly without reasoning too much like that for hyperkalemia I saw it’s typical and I knew what he had / in that sense it’s easier*” (participant no. 14)
	“*here there was precordialgia = thoracic pain = PIED pericarditis-infarction-pulmonary embolism-dissection [there was a] ST segment anomaly so it can only be infarction or pericarditi*s” (participant no. 16)
Inductive coding	
Thematic categories	Representative quotes
Panel members identity	“*they tell us cardiology specialists, but we don’t know who they are at all*” (participant no. 2) *–* “*The thing is, we know cardiologists, who… you know… [do a bad job] […] so, people we don’t trust completely*” (participant no.1)
No scoring	*“Now that we are residents, we don’t to be judged […] we do it because we want to, not because we’re forced to”* (participant no.5)
Uncertainty	“*they don’t all have the same opinion and that doesn’t mean that if we don’t all do the same […] it doesn’t necessarily mean that we’re wrong I found that these moments when there were different opinions, between the cardiologists as well as between the general practitioners, were a little less demeaning than the MCQ methods which it’s right it’s wrong and that’s it*”

### Deductive coding

The qualitative analysis comprised 203 codes in the focus groups and 206 codes in the individual interviews, which were divided into five groups based on the components of reflection in the Nguyen model: thoughts and actions; attentive, critical, exploratory, and iterative process (ACEI); underlying conceptual frames component; change and self (see Table 3).

**Table 3:** deductive coding tree

Themes	Number of codes in focus groups	Number of codes in elicitation interviews
**ACEI component**	79	65
Attentive interactions	31	26
Critical interactions	25	16
Exploratory interactions	10	9
Iterative interactions	13	14
**The underlying conceptual frames component**	0	53
**Thoughts and actions component**	36	38
**The Self component**	63	33
**The view on the Change component**	25	17
Total	203	206

**Table:** 

**ACEI**	65
A	26
C	16
E	9
I	14
**CC**	**53**
CC	53
**PA**	**38**
PA	38
**S**	**33**
S	33
**VC**	**17**
VC	17

#### The “thoughts and actions” component

Participants felt the need to train themselves to read ECGs based on their upcoming confrontation with ECGs during the internship and their fear of error.

*“So I said to myself that for the emergency unit internship, I had to reread it beforehand because if something happens in the middle of the night, I would finally be able to handle the situation*” (participant no. 12).

“*About the ECG, since it’s normal, I don’t do anything, but obviously you reassure [the patient]. Of course, right? Now [during the internship], obviously, you always reassure people, you don’t even realize it, actually*” (participant no. 3).

### The ACEI component

Many codes derived from the analysis of the interview transcripts correspond to an attentive, critical, exploratory, and iterative thought process:

“*I would put myself in the situation; I’d tell myself, I’m the doctor, this patient is my patient, and yeah, I was truly totally in the case*” (attentive interaction – participant no. 8);

*“As I saw that I was missing something each time, I tried to be more systematic and to read them properly”* (critical interaction - participant no. 12),

“*I found it interesting that normal ECGs were included as well, especially to make us think about the limits of normality, which is something we do not see enough of in training courses*” (exploratory interaction – participant no. 11).

“*Well, I adjusted to the answers, anyway – for instance, about reassurance, there was a first case where I hadn’t written it down, and in the answer, he put reassurance, and so in the last case where it was anxiety and all that, I put reassurance*” (iterative interaction – participant no. 9).

Some aspects could be improved to foster the process of reflection. In particular, the long duration and cognitive overload may have had negative impacts on learning: “*In fact, it was very long, and at one point I could not manage; I was confusing all the ECGs I saw*” (attentive interaction – participant no. 12) - “*I believe that the training is planned to last for three hours; I find that three hours, I find that it is tight if we want to pause on the resources to go deeper*” (exploratory interaction - participant no. 11).

Critical interaction was sometimes limited by the difficulty of understanding the panel answer: *“when it is a little too technical [the cardiologist interpretation of the ECG], I can quite easily let it go and say to myself that it is too much for me”* (critical interaction - participant no. 15).

### The change component

Several utterances made by participants illustrated a desire to change their thought processes: “*for instance, you see [atrial fibrillation] […] I told myself, ‘Okay, we have the right to handle that ourselves’*” (participant no. 1).

“*I made sure to do things a little bit more seriously, because, you know, there are actually important things to monitor, so yeah, that and being thorough when I read my ECGs*” (participant no. 4) – “*Same here, yeah, it’s true that we often tend to just glance at the thing; maybe we don’t necessarily, you know* [laughs], *do it as well as we did when we were students*” (participant no. 7) – “*do a better job of reading the ECG*” (participant no. 5).

### The underlying conceptual frames component

Our analysis showed elements in the individual interviews that were related to the underlying conceptual frames of the participants. Participants noted that the program caused them to consider their clinical reasoning processes in light of the clinical cases. They focused on their nonanalytical clinical reasoning strategies: “*it is true that for hyperkalemia, it is a typical picture to recognize directly without reasoning too much like that for hyperkalemia; I saw it is typical, and I knew what he had/in that sense it is easier*” (participant no. 14). The more typical the clinical picture was, the more oriented the ECG reading was. The participants sought specific abnormalities on the ECG and then ruled out some hypotheses based on their findings. These nonanalytical clinical reasoning strategies were often the source of their errors. Therefore, participants decided to rely more on analytical clinical reasoning strategies: “*To interpret […] the first ones I did like that [a global interpretation], in fact, I checked whether there was something obvious afterward as I saw that I was missing something each time. I tried to be more systematic and to read them well*” (participant no. 12).

Regarding analytical clinical reasoning strategies, some participants used a systematic reading method: “*I remember in the cardio teaching when I was a graduate student, he [the teacher] said that you had to look at all the [ECG leads] because you could be surprised, and it pushed me back to looking properly to be more regular* [during the LbC ECG training]” (participant no. 12). In this type of reading, the participants relied on their clinical scripts: “*here, there was precordialgia = thoracic pain = PIED pericarditis-infarction-pulmonary embolism-dissection; [there was a] ST segment anomaly, so it can only be infarction or pericarditi*s” (participant no. 16).

When participants faced issues related to clinical reasoning, they used resources during the training to develop or create new prototypes and clinical scripts: “*When I saw the answer to each question, I looked again on the internet […] I looked to see whether there were more examples of the same type to have additional images in head*” (participant no. 13). Some participants kept these resources to foster a change in their future practice. The main limitation to such change mentioned by participants was the lack of explicitness in the interpretation of the panelists: “*I thought it was good to have the opinion of the cardiologist or the general practitioner each time, but I found that it was not always detailed in relation to how we see that it is a Bouveret or a Brugada; I found that it was a little lacking* [in details]” (participant no. 13).

Finally, clinical reasoning strategies were also guided by emotional factors and uncertainty: “*In this situation, for example, I would send [hesitates a lot] […] it would have been about the complaint […] I would have sent him to the emergency room, with regard to the ECG; I do not know […] We are still worried*” (participant no. 12).

### The self component

The participants easily connected their thoughts to their ‘selves’ both during and after the training program: “*My way of reasoning is fairly logical compared to the others; I’m a little bit reassured, and I’ve gained a little bit of trust in myself*” (participant no. 3). This approach may have encouraged some of them to use the ECG in their future practice: “*honestly, I didn’t think I would need to read ECGs afterward [after finishing his or her studies] but in fact there are plenty of GPs who have an ECG, and it’s not bad; it allows a first screening*” (participant no. 12).

The absence of grades and the purely learning-oriented goal of the training program was important with regard to this component: “*I think it isn’t bad doing it like this because in the end, when I did it, I wasn’t under any pressure, and so you know, you’re not stressed out. When you’re being graded or evaluated, you get stressed out, whereas this is truly just for us*” (participant no. 5).

### Inductive coding

Inductive coding revealed interesting thoughts that emerged in this LbC format concerning the impact of the identity of the panel members, the absence of a scoring system and the question of uncertainty in ECG reading.

Participants were unsure of what value they should attribute to the answers offered. This uncertainty seems to foster a critical distance from the role model that recognized panel members could provide: “*they tell us cardiology specialists, but we don’t know who they are at all [in the ECG LbC training program panel]*” (participant no. 2) *–* “*The thing is, we know cardiologists, who… you know… [do a bad job] […] so, people we don’t trust completely [the cardiologists panel]*”. These parts were also coded as critical thoughts in the ACEI component.

The absence of a scoring system and the assessment of the learning goal of the training program was important to the participants: “*I think it isn’t bad [the ECG LbC training program] doing it like this, because in the end, when I did it, I wasn’t under any pressure, and so you know, you’re not stressed out. When you’re being graded or evaluated, you get stressed out, whereas this is truly just for us*” (participant no. 5). This part was also coded as an attentive thought associated with the ACEI component.

Uncertainty is often a source of stress in the context of ECG interpretation in real-life situations for participants and a potential obstacle to the use of ECGs in their future practice. The participants highlighted three main elements of this LbC program that facilitated more effective management of this uncertainty. First, the existence of a panel of experts, whose interpretations varied slightly but which led in all cases to an adapted management of the situation: “*they do not all have the same opinion, and that does not mean that if we do not all do the same […] it does not necessarily mean that we’re wrong. I found that these moments when there were different opinions among the cardiologists as well as among the general practitioners were a little less demeaning than the MCQ [multiple choice question] methods, which are it is right or it is wrong and that’s it*” (participant no. 11).

Second, situations featuring normal or subnormal ECGs are an important source of uncertainty for the participants, and they felt the need to train on these situations: “*finally, when I’m least sure of myself is when it is normal/and in that sense, it would be interesting to do more subnormal or physiological ECG*” (participant no. 15).

Finally, participants emphasized the use of transversal skills to manage a patient situation based on the suggestions made by the general practitioners panel: “*it allowed me to better conceptualize the call to the cardiologist, for example, to better understand when we let the patient make an appointment or when I must call. It allowed me to touch upon this notion of temporality, a short term, a long term*” (participant no. 11).

## Discussion

Based on the model discussed in Nguyen et al. [9], analysis of the data collected by this study demonstrates the presence of all components of reflection. During and after the LbC program, participants appear to have engaged in ECG interpretations that were characterized by attentive, critical, exploratory, and iterative interactions between their thoughts and actions. These interactions were change-oriented.

These findings are consistent with those of Fernandez et al. from 2016 [1]. They tend to support the notion that LbC sessions promote the development of reflexivity among students. In 2023, Fernandez et al. argued that instructional design in LbC strengthened appropriate reflexive skills by selecting cases that involve a significant degree of uncertainty [7]. In our training, uncertainty was consistently present due to the diversity of interpretations from the panel of cardiologists or the proposed management options from the panel of general practitioners.

Learning by concordance could thus either actually increase reflection among learners [1] or at least facilitate other metacognitive thought processes [22]. As reflection must be viewed as a continuum rather than a dichotomic process [23–27], these data suggest that the specificities of LbC could favor the development of reflection [1].

Two methodological choices appear to deserve consideration in cases in which the objective of an LbC program is to boost reflection among learners: the anonymity of panel members and the calculation of a score. While the identity of panel members is usually announced at the beginning of the proceedings [1–4], not disclosing their identity appears to have reinforced the ACEI component of reflection in our study. Other studies should investigate the importance of this parameter, but we advise trainers to reflect on this aspect when developing an LbC tool. Similarly, while a score is actually calculated after each question or upon completion of the LbC [1, 2, 28–30], our choice to refrain from using scores also appears to have reinforced the ACEI component. This choice renders learner self-assessment the only form of evaluation in the program. We believe that this approach is consistent with the choices recommended in competency-based approaches and should facilitate the inclusion of LbC in competence-based curricula [31–34].

In addition, this type of LbC program might increase learners’ tolerance and ability to deal with uncertainty. As acknowledging the feeling of uncertainty helps learners address it, the expression of this feeling by students and panelists should be encouraged during the LbC program [35]. Researchers are increasingly highlighting the fact that the ability to deal with uncertainty is a major goal of medical education, as intolerance of uncertainty can lead to burnout, ineffective communication strategies, cognitive biases, and inappropriate resource use [36].

Such an LbC program on ECG interpretation may help students develop the ability to self-regulate their learning, which is necessary for their continual professional development [37]. This finding is consistent with the societal expectations currently placed on medical schools [38].

There are limitations to this study. The principal limitation can be phrased as a question : “how can we prove that an intervention fosters reflection?”. We tried to find components of reflection by reference to qualitative data collected through focus groups and elicitation interviews. However, how can we differentiate between program-related reflection and our data collection-induced reflection? We tried to mitigate potential bias by asking specific questions in our guides that were inspired by previous studies, but part of the reflection we highlighted might ultimately have been the result of our data collection processes [15]. Another limitation of this research is the delay between the training and the focus groups. It is possible that the reflective process subsequently verbalized by the participants is not truly the process that was used at the time of the training.

Nevertheless, this study is the first to describe a training program combining the LbC principles of perception and reasoning. It provides arguments that support the use of LbC in the education of health care professionals since uncertainty is a prominent feature of their work and reflection is a desired feature.

## Conclusions

This study supports the claim that the use of LbC in the context of ECG interpretation programs could foster reflection in future general practitioners. As in Nguyen’s definition, participants engaged in attentive, critical, exploratory, and iterative interactions with their thoughts and actions, as well as their underlying conceptual frameworks, with a view to changing them and a view on the change itself in an LbC training program on ECG interpretation. Health science educators looking to cultivate reflection among their students may consider incorporating LbC into their training programs.

Subsequent studies could assess and compare the impact of various instructional designs on the development of reflection with LbC, such as the absence of scoring or the anonymity of members in the reference panel.

### Electronic supplementary material

Below is the link to the electronic supplementary material.

Supplementary Material 1: Example of a clinical case of the LbC program

Supplementary Material 2: Interviews guides

## Data Availability

The datasets used and analyzed during the current study are available from the corresponding author on reasonable request.

## Abbreviations

LbC: Learning-by-Concordance
ECG: electrocardiogram
ACEI: attentive, critical, exploratory, and iterative processes
GPD: general practice department
CNIL: French National Commission on Information Technology and Liberties
MCQ: multiple choice question
