# Supplementary material for: Impact of an online learning by concordance program on reflection
Source: BMC Med Educ. 2023 Nov 1;23:822. doi: 10.1186/s12909-023-04799-9 (PMC10621083; doi:10.1186/s12909-023-04799-9)
Supplement: Supplementary file 1 — Supplementary Material 1: Example of a clinical case of the LbC program [file 12909_2023_4799_MOESM1_ESM.docx]

*Supplementary material 1 - Example of a clinical case of the LbC program - first page*

You are seeing Mr. Gross for a consultation. He is a 65-year-old retired individual and a former French teacher whom you know well for his strong opinions on the relative usefulness of medications. Currently, he is taking Amlodipine 10mg and Ramipril/Hydrochlorothiazide 5mg/12.5mg once a day.

Mr. Gross informs you of his decision to take up running again, which, according to him, is "a much better medicine than those from pharmaceutical industries."

During the examination, you observe a blood pressure reading of 170/105mmHg with no other clinical abnormalities. You decide to perform an ECG.


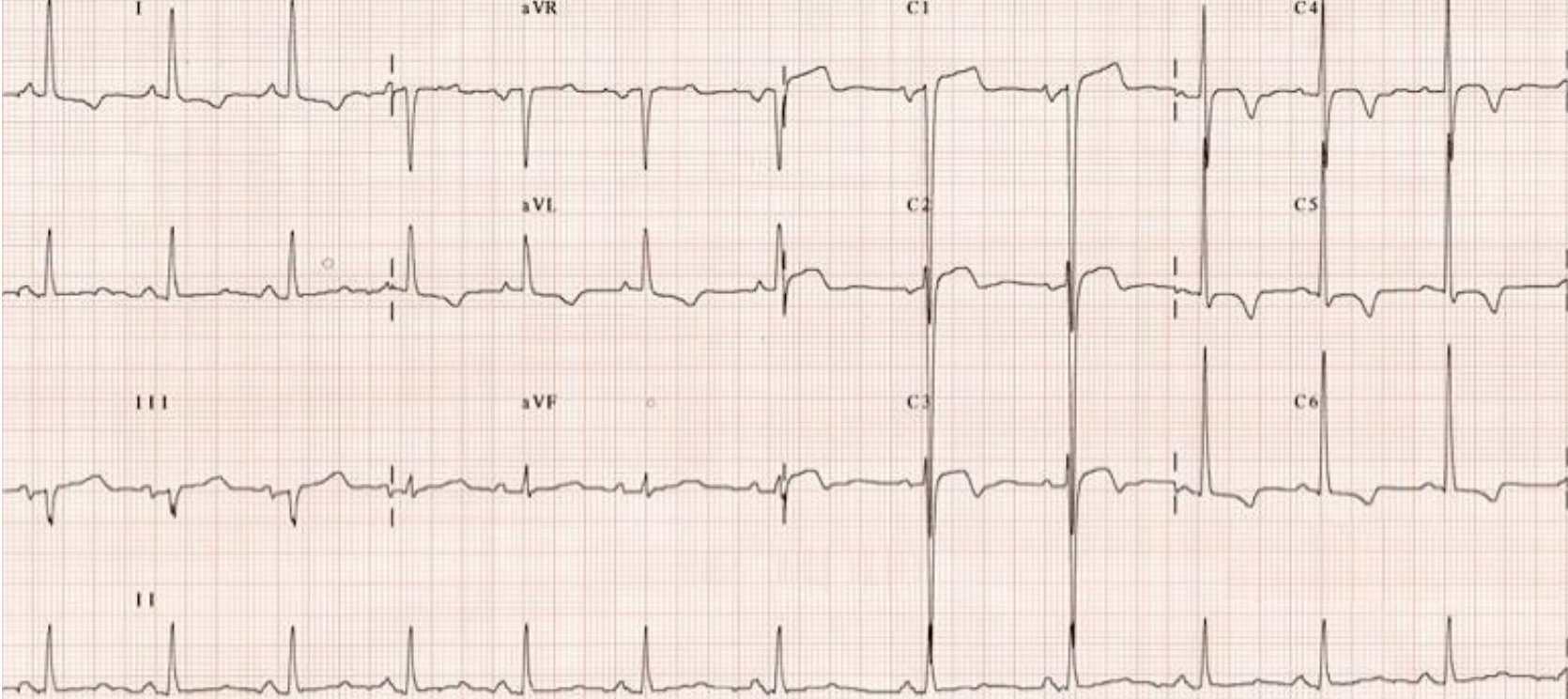


What does the interpretation of this ECG tracing suggest to you? Justify your response.

*Second page*

Your response was [student’s response]

The responses from the panel of cardiologists are as follows:

1. Signs of left ventricular hypertrophy. Hypertensive hypertrophic cardiopathy? Sinus rhythm (Cardiologist #1).

2. Signs of left ventricular hypertrophy. Regular sinus rhythm, normal electrical axis, broad QRS in V5-V6, and deep S waves in V1-V2 with related repolarization anomalies (negative T waves in V4-V6 and D1-aVL, as well as slight ST-segment elevation in V1-V3) (Cardiologist #2).

3. Systolic left ventricular hypertrophy. Sokolow criteria and signs of lateral overload, no ischemia (Cardiologist #3).

4. Left ventricular hypertrophy in probable hypertrophic cardiomyopathy. Sinus ECG with an increased Sokolow index (SV or V+ RV), ST-segment elevation in V1-V2-V3, and asymmetric negative T waves (Cardiologist #4).

What would be your approach in this situation in a general practice setting?

*Third page*

Your response was [student’s response]

The responses from the panel of general practitioners are as follows:

- No running due to the risk of acute ischemia, moderate regular walking, and reinforcement of antihypertensive treatment. Cardiac ultrasound to assess the impact of inadequate treatment (General Practitioner #1).

- Referral to a cardiologist for a cardiac ultrasound. No issuance of a medical certificate of non-contraindication before the cardiologist's opinion is obtained. Simultaneously, inquire about the patient's treatment adherence (General Practitioner #2).

- I would ask him to undergo a stress test before considering resuming physical activity. Reconditioning to exercise would be beneficial for him. I would discuss medications with him, particularly what can be expected from them, possibly using a risk calculation software (General Practitioner #3).

Summary:

In the face of this patient with poorly controlled hypertension and cardiac involvement (signs of left ventricular hypertrophy on ECG), resuming physical activity is not recommended without promptly consulting a cardiologist. Additionally, this consultation could be an opportunity to discuss the patient's adherence to antihypertensive treatments and potentially supervised exercise retraining.

References:

1. Management of hypertension in adults - Memo sheet, September 2016 - HAS (French National Authority for Health).
