# Supplementary material for: Impact of an online learning by concordance program on reflection
Source: BMC Med Educ. 2023 Nov 1;23:822. doi: 10.1186/s12909-023-04799-9 (PMC10621083; doi:10.1186/s12909-023-04799-9)
Supplement: Supplementary file 2 — Supplementary Material 2: Interviews guides [file 12909_2023_4799_MOESM2_ESM.docx]

*Supplementary material 2 - interviews guides*

*Focus group:*

1/ Presentation of the participants and investigators

2/ How did you hear about this training?

3/ How did the training go? What involvement did you have?

4/ What did you think about this training?

5/ What did you learn?

knowledge?

Contextual?

Behavior?

Details about ECG?

6/ What did you think about? How did you do this?

7/ After your training, how did you integrate those learnings into your practice?

8/ Results: how has your practice changed?

Is there an assessment? a reaction of your supervisor?

9/ What did you think about the uncertainty in this training?

*Interview guide - Elicitation interview:*

1/ Introduction: presentation, consent, confidentiality, goal

2/ General elicitation questions:

- Could you tell me about a time when you did an ECG before this training?

- What made you choose this training?

- I suggest - if you agree - to take the time to let the moment when you did the ECG training come back...

3/ Questions related to randomly picked vignettes:

- When faced with this situation (vignette), what did you think?

- How did this vignette unfold?

- What did you use for solving this vignette? (to be discussed based on the response to the question at the beginning of the interview “Could you tell me about a time when you did an ECG before this training”, especially if there is a difference in response)

- Could you detail the steps you took to solve this vignette?

- How did you go about solving this vignette?

4/ Questions about the training as a whole:

- How did the succession of vignettes unfold?

- What does this training change for your practice? What do you take away from this training to read an ECG in the future?
